# Usability Evaluation of Dashboards: A Systematic Literature Review of Tools

**DOI:** 10.1155/2023/9990933

**Published:** 2023-02-22

**Authors:** Sohrab Almasi, Kambiz Bahaadinbeigy, Hossein Ahmadi, Solmaz Sohrabei, Reza Rabiei

**Affiliations:** ^1^Department of Health Information Technology and Management, School of Allied Medical Sciences, Shahid Beheshti University of Medical Sciences, Tehran, Iran; ^2^Digital Health Team, Australian College of Rural and Remote Medicine, Brisbane, QLD, Australia; ^3^Medical Informatics Research Center, Institute for Futures Studies in Health, Kerman University of Medical Sciences, Kerman, Iran; ^4^Centre for Health Technology, Faculty of Health, University of Plymouth, Plymouth PL4 8AA, UK

## Abstract

**Introduction:**

In recent years, the use of dashboards in healthcare has been considered an effective approach for the visual presentation of information to support clinical and administrative decisions. Effective and efficient use of dashboards in clinical and managerial processes requires a framework for the design and development of tools based on usability principles.

**Objectives:**

The present study is aimed at investigating the existing questionnaires used for the usability evaluation framework of dashboards and at presenting more specific usability criteria for evaluating dashboards.

**Methods:**

This systematic review was conducted using PubMed, Web of Science, and Scopus, without any time restrictions. The final search of articles was performed on September 2, 2022. Data collection was performed using a data extraction form, and the content of selected studies was analyzed based on the dashboard usability criteria.

**Results:**

After reviewing the full text of relevant articles, a total of 29 studies were selected according to the inclusion criteria. Regarding the questionnaires used in the selected studies, researcher-made questionnaires were used in five studies, while 25 studies applied previously used questionnaires. The most widely used questionnaires were the System Usability Scale (SUS), Technology Acceptance Model (TAM), Situation Awareness Rating Technique (SART), Questionnaire for User Interaction Satisfaction (QUIS), Unified Theory of Acceptance and Use of Technology (UTAUT), and Health Information Technology Usability Evaluation Scale (Health-ITUES), respectively. Finally, dashboard evaluation criteria, including usefulness, operability, learnability, ease of use, suitability for tasks, improvement of situational awareness, satisfaction, user interface, content, and system capabilities, were suggested.

**Conclusion:**

General questionnaires that were not specifically designed for dashboard evaluation were mainly used in reviewed studies. The current study suggested specific criteria for measuring the usability of dashboards. When selecting the usability evaluation criteria for dashboards, it is important to pay attention to the evaluation objectives, dashboard features and capabilities, and context of use.

## 1. Introduction

Nowadays, healthcare organizations encounter various forms of information chaos, such as information overload, erroneous information, scattered information, and incompatibility of information with job requirements [1]. Meanwhile, effective and efficient use of data in managerial and clinical decision-making can be complicated because of the massive amount of data, data collection from various sources, and lack of data organization, which can lead to increased errors [2], delayed service delivery [3], and patient safety risks [4]. Agile healthcare organizations use relevant data in their daily operational decisions, ranging from supply chain management and staff planning to care delivery planning and community health management [5].

Healthcare systems are increasingly using business intelligence systems for monitoring performance indicators [5]. According to Loewen and Roudsari, these systems are used for collecting, analyzing, and presenting organizational data to intended users in their required format in line with meeting organizational objectives [6]. Dashboards are one of these systems widely used in the healthcare settings. Through data visualization, dashboards provide practical feedback to improve performance, promote the use of evidence-based methods, and enhance workflow and resource management [7, 8]. These tools also use visual representations, such as charts and color coding, to facilitate the interpretation of information [8, 9].

Generally, dashboards, as data management tools, collect data from various information systems and present them based on key performance indicators in a concise, comprehensive, meaningful, and intelligent manner. Additionally, dashboards provide useful information to managers to enable them to check their performance at a glance, easily identify the existing problems and their leading causes, and take necessary actions for performance improvement [10, 11]. Nevertheless, development of dashboards is a complex process, as the information needs of users are completely dependent on the context of use and factors, such as clinical environment, occupational roles, and patient population, which also influence the selection of proper data elements, visualizations, and interactive capabilities [12–14]. Therefore, in the design of dashboards, particular attention must be paid to usability principles and human factors to deliver interactive and data sharing capabilities [15].

In order to have efficient dashboards for clinical and managerial decisions, these tools should have no or minor usability problems. One of the methods to ensure the proper design of software programs and health information systems, such as dashboards, is to use proper evaluation criteria for system usability. Generally, usability evaluation deals with various software features, including the ease of learning, efficiency, ease of use, memorization, error prevention, and user satisfaction. According to the ISO 9241-11, usability can be defined as “the extent to which a product can be used by specified users to achieve specified goals with effectiveness, efficiency and satisfaction in a specified context of use” [16]. This definition refers to the user's experience of human-machine interactions. Regardless of the product type, it is not only important to achieve specific goals but also the user's satisfaction and experience of the system are significant [16]. For dashboards, similar to other information systems, usability can be defined as “the extent to which a system is used by users to achieve specific goals with high efficacy, efficiency, and satisfaction” [17].

One of the most well-known classifications for usability evaluation methods was developed by Nielsen [18] and Holzinger [19]. According to this classification, the usability evaluation methods can be divided into two categories: usability inspection and usability testing. The first category refers to experts' inspections of the user interface design based on standards using inspection techniques. On the other hand, usability inspection is aimed at identifying the usability problems of a design [20], although it can be also applied to determine the user interface characteristics of systems that have not been implemented. The main methods of usability inspection include (1) heuristic evaluation, (2) cognitive walkthrough, and (3) action analysis [21].

The process of usability testing is different from that of usability inspection. In usability testing, several end users, on behalf of other users, implement a series of tasks using a prototype system so that experts can detect usability problems by observing their performance. These methods can provide direct access to information on how users employ systems [19]. Some of the most common usability testing methods include (1) paper and pencil tests, (2) think aloud, (3) codiscovery, (4) field observation, (5) query techniques, (6) questionnaires, and (7) card sorting [21].

Questionnaires have been employed as usability testing methods to collect the users' demographic data and opinions [22]. In recent years, various questionnaires have been developed to evaluate the usability dimensions [22]. The most well-known questionnaires for usability testing include the Computer System Usability Questionnaire (CSUQ), Post-Study System Usability Questionnaire (PSSUQ), Questionnaire for User Interaction Satisfaction (QUIS), Software Usability Measurement Inventory (SUMI), System Usability System (SUS), Usability Metric for User Experience (UMUX and UMUX-Lite), and Usefulness, Satisfaction, and Ease of Use (USE) [21, 22].

Our search indicated that the questionnaires used for the usability evaluation of dashboards are not specially designed for this purpose, and they could fail to appropriately measure the main capabilities and features of these systems.

On the other hand, previous studies mainly have focused on identifying important functional and nonfunctional requirements of healthcare dashboards [8, 9], the effect of dashboards in improving patient outcomes and in healthcare provider satisfaction [12, 17], and developing frameworks for designing dashboards [13].

Given the role of dashboards in the decision-making process and the multiplicity of questionnaires, it can be challenging to select a proper questionnaire for the usability evaluation framework of dashboards. Since no study has yet presented a framework or tool for evaluating the usability of dashboards, the present study is aimed at reviewing the existing questionnaires for the usability evaluation of dashboards and at providing appropriate criteria for such assessments.

## 2. Methods

### 2.1. Data Sources and Search Strategy

The search and data extraction stages were performed based on the PRISMA checklist [23]. Articles were extracted by searching the PubMed, Web of Science, and Scopus databases. A combination of MeSH terms and keywords related to dashboards, usability, and questionnaires was used for the search strategy (Table 1). The final search of articles was carried out without any time restrictions. Two researchers (SA and SS) searched and retrieved articles independently, and any disagreement was discussed with the senior author (RR).

### 2.2. Inclusion and Exclusion Criteria

#### 2.2.1. Inclusion Criteria

The inclusion criteria were as follows: (1) English articles published on the design, implementation, and evaluation of dashboards in healthcare settings, including clinics, hospitals, or any healthcare center providing services for disease prevention, treatment, rehabilitation, and medical education and (2) the use of questionnaires for evaluating dashboards.

#### 2.2.2. Exclusion Criteria

The exclusion criteria were as follows: (1) non-English studies, (2) focusing on only dashboard design or dashboard evaluation, (3) use of evaluation methods other than questionnaires to evaluate usability, and (4) lack of access to the full text of articles.

### 2.3. Study Selection, Article Evaluation, and Data Extraction

In the study selection phase, two authors (SS and SA) performed screening, selection, and full-text review and two authors (KB and HA) performed qualitative evaluations of papers; any disagreement was checked and eliminated through discussing with the senior author (RR). The quality of each study was checked by using the Joanna Briggs Institute (JBI) critical appraisal tools. The JBI-MAStARI instrument was used for RCT and quasiexperimental studies (nonrandomized experimental studies) [24]. For RCT studies, there is a checklist containing 13 questions with four options (“yes,” “no,” “unclear,” and “not/applicable”). For quasiexperimental studies, there is a checklist covering 9 questions with four options (“yes,” “no,” “unclear,” and “not/applicable”).

One score was assigned for each “yes” answer, and in case 70 of the questions led to “yes” answer, the risk of bias was considered as low. The risk of bias was regarded as “moderate” in the event of obtaining 50-60% of “yes” answers. Ultimately, a “high-risk” bias was assigned to “yes” responses below 50% (Appendix A Table A1 and Appendix A Table A2).

For data extraction, the features of questionnaires, including the number and scoring of questions, criteria, and reliability, were first investigated (Table 2). Next, the year of the study, country of the study, evaluation criteria for dashboards, and questionnaires used for the evaluation of dashboards were extracted for each article and entered into Microsoft Excel for analysis (Appendix B Table A3). Moreover, for data extraction, the questionnaires were assessed, and the evaluation criteria for dashboards were extracted (Table 3). The reasons for selecting or removing each criterion for dashboard evaluation in the questionnaires are presented (Appendix C Table A4).

## 3. Results

**Figure 1:**
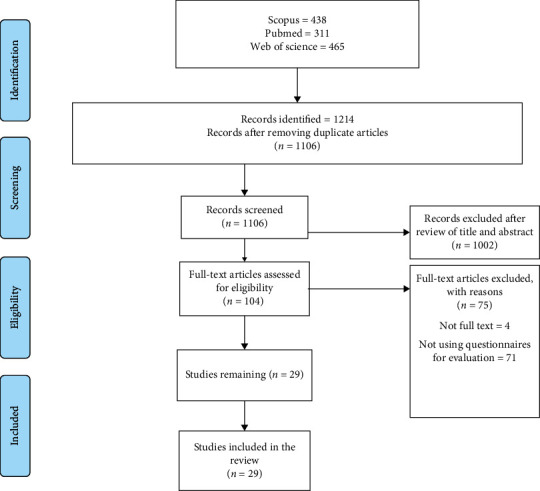
The study flow diagram based on the PRISMA guidelines.

A total of 1214 articles were retrieved after searching the databases. Using EndNote software, 108 duplicate articles were removed, and 1106 articles remained. After reviewing the titles and abstracts of studies, 1002 articles were removed, and 105 articles remained. Finally, by reviewing the full text of studies, 75 articles were removed, and 29 articles were included in the present study. The article selection process is presented in Figure 1.

### 3.1. Quality Assessment

Based on the qualitative evaluation of articles using the Joanna Briggs Institute (JBI) appraisal tool, among nonclinical studies, 8 (31%) articles were classified to have “moderate” qualitative evaluations for dashboards, while 18 (69%) articles were placed in the “low-risk group” (Appendix A Table A1). Additionally, three clinical trials were evaluated using the JBI tool, all of which were placed in the low-risk group (Appendix A Table A2).

### 3.2. General Characteristics of Studies

**Figure 2:**
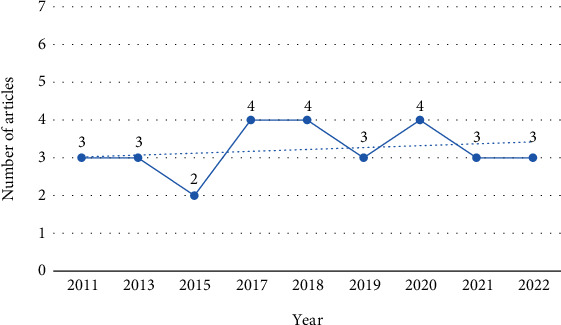
Number of publications by year.

According to our review of selected studies, 29 (89%) articles, including 23 cross-sectional studies, three case report studies, one longitudinal study, and three experimental and clinical trials (11%), were found to be descriptive. As shown in Figure 2, the number of articles focusing on dashboards in healthcare is increasing. Concerning the location of studies, the majority of studies were conducted in the United States (39%), England (14%), Germany (7%), and South Korea (7%), respectively.

**Figure 3:**
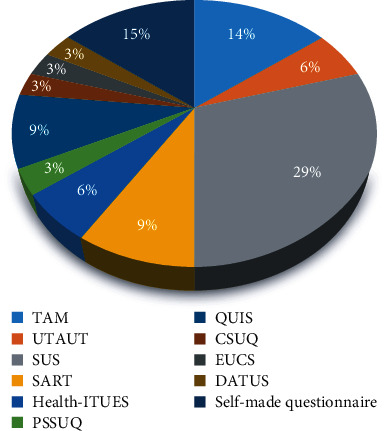
Number of questionnaires used in previous studies.

Five studies used researcher-made questionnaires, while 24 studies used existing questionnaires. In five studies, two questionnaires were used to evaluate dashboard usability. The most widely employed questionnaires were the System Usability Scale (SUS), Technology Acceptance Model (TAM), Situation Awareness Rating Technique (SART), Questionnaire for User Interaction Satisfaction (QUIS), Unified Theory of Acceptance and Use of Technology (UTAUT), and Health Information Technology Usability Evaluation Scale (Health-ITUES), respectively (Figure 3).

### 3.3. Usability Evaluation Criteria for Dashboards

According to the review of other questionnaires used in previous studies (Table 3), the following criteria were identified for dashboard evaluation: usefulness, operability, learnability, ease of use, suitability for tasks, improvement of situational awareness, satisfaction, user interface, content, and system capabilities.

#### 3.3.1. Usefulness

Usefulness is usually defined as meeting a customer's needs or providing a competitive advantage with the product's attributes or benefits. Designers, generally, aim to deliver useful products. In the reviewed studies, the “usefulness” criterion was used instead of “effectiveness and efficiency” and it was used in four questionnaires, including the Health-ITUES, PSSUQ, CSUQ, and TAM, to evaluate the usability of dashboards.

#### 3.3.2. Operability

It refers to a user's ability to use and control a dashboard for performing their tasks. In the present study, operability included criteria, such as representation of data in detail, access to various filters and reports, and ability to correct errors and support user. The user control is measured under the “operability” criterion.

#### 3.3.3. Learnability

Learnability is a quality of software interface that allows users to quickly become familiar with them and able to make good use of all their features and capabilities.

#### 3.3.4. Ease of Use

It is a fundamental concept explaining how easily users can employ a dashboard. This criterion was used for dashboard evaluation in the EUCS, Health-ITUES, and TAM questionnaires.

#### 3.3.5. Suitability for Tasks

This criterion can help to assess if users can find out whether a product or system is appropriate for their needs. It provides support for the users' daily activities and ensures the compatibility and organization of data on the screen with the user's tasks.

#### 3.3.6. Improvement of Situational Awareness

Situation awareness at a fundamental level is about understanding what is going on and what might happen next. The criteria for evaluating situational awareness were divided into instability representation, complexity representation, variability representation, arousal support, concentration support, spare mental capacity support, and division of attention.

#### 3.3.7. Satisfaction

This criterion refers to satisfaction with the features, capabilities, and ease of use of a dashboard.

#### 3.3.8. User Interface

It consists of visual and interactive tools. Visual tools in a dashboard involve color coding for data visualization, histogram plots, pie charts, bar graphs, gauges, data labels, and geographic maps. The interactive techniques also include customizable searching, summary view, drill up and drill down, data ordering and filtering, zoom in and zoom out, and real-time feature.

#### 3.3.9. Content

This criterion involves the quantity and quality of data displayed by a dashboard. The quantity of displayed data was measured using two questionnaires (SART and PSSUQ), while quality was measured using SART. The amount of displayed data and their compatibility with the users' tasks were also evaluated, and data accuracy, timeliness (being up-to-date), comprehensiveness, and relevance were used for measuring data quality.

#### 3.3.10. System Capabilities

Evaluation of compatibility is a criterion to assess software in terms of compatibility with work-related requirements. The dashboard capabilities are evaluated to determine how well its compatibility to work-related processes and how well it satisfies the users' data requirements.

## 4. Discussion

In the present study, questionnaires used in previous research were reviewed to suggest criteria for dashboard evaluation. Generally, questionnaires are the most commonly used tools for usability evaluation because of the simplicity of data analysis [53, 54]. According to the findings, although SUS does not cover the efficiency, memorability, or error criteria and consists of a series of general questions for usability evaluation [55], it was the most widely used tool for dashboard evaluation. In four studies, SUS was used along with other questionnaires for dashboard evaluation [32–35].

In the study of Hajesmaeel-Gohari et al., the SUS questionnaire was the most used tool for measuring usability [56]. In the study of Sousa and Dunn Lopez conducted with the aim of identifying the questionnaires used for usability evaluation of electronic health tools, the main used criteria in the investigated questionnaires included learnability, efficiency, and satisfaction. The memorability was the least used criterion [57].

In the present study, “satisfaction” and “learnability” were proposed as two key criteria for evaluating the usability of the dashboards, and “efficiency” was also proposed as one of the subcriteria of “usefulness.” One criterion, i.e., “memorability,” was not included in the proposed framework, as the learnability could cover the required metrics.

To take advantage of usability evaluation tools, it is important to pay attention to the study objectives, used technologies, and context of use [53, 58, 59]. The ISO/IEC 25010 consists of suitability for tasks, learnability, operability, user error protection, user interface aesthetics, and accessibility [60]. The ISO/IEC 9241-11 also suggests measure such as effectiveness, efficiency, and satisfaction for usability evaluation [60]. Additionally, Nielsen's criteria were used for evaluating dashboard including efficiency, memorability, error, learnability, and satisfaction [61]. In the current study, usefulness was used rather than the effectiveness and efficiency criterion, and it was used in four questionnaires, including the Health-ITUES, PSSUQ, CSUQ, and TAM.

In general, TAM and UTAUT are the most widely used acceptance models in health informatics because of their simplicity, and these mainly focus on the usefulness and easy to use technology [56].

The dashboard “operability” criterion in the current study refers to the user's ability to the user's control over the software, error correction ability, and quick recovery. In addition, in previous studies, the “operability” criterion referred to error correction, error correction in use, default value availability in use, message understandability, self-explanatory error messages, operational error recoverability in use, and time between human error operation in use [62]. Moreover, improvement of situational awareness was considered as one of the evaluation criteria for dashboards. Overall, dashboards provide key data that should be monitored effectively to be notified of what is occurring in one's work environment. The results of previous studies indicated that dashboards have the potential to accelerate data collection, decrease the cognitive load, reduce errors, and improve situational awareness in healthcare settings [8, 16].

Additionally, the “user interface” criterion includes what a user uses to interact with the system. Some interface hardware components include a keyboard, mouse, microphone, and user interface (e.g., graphic forms, language tools, and interactive tools) [22]. With respect to the user interface of dashboards, the application of visual and interactive features was suggested in the present study, considering data representation and interactive visualization as critical features [63]. Visualization systems, such as dashboards, are capable of two main functions: representation and interaction [64]. Besides interactive features, it is also essential to consider the visual features for an effective and understandable representation of indicators, which can lead to an effective interaction with data and instantaneous monitoring of performance indices [61, 65]. In Shneiderman's study, interactive features included overview, zoom, filter, details-on-demand, relate, history, and extraction [66]. In addition, interactive techniques in M. Khan and S. Khan's study included zoom and pan, overview and detail, and filtering [67].

In the current study, the quantity and quality of data represented by dashboards were considered as the content criteria. In the EUCS questionnaire, being up-to-date is considered as a separate criterion for dashboard evaluation, while being up-to-date, accurate, comprehensive, and relevant were considered as data quality features in previous research [68, 69]; consequently, in the present study, these features were considered for data quality. Data quality refers to data integrity, data standardization, data granularity, and data completeness, which are essential for a well-designed dashboard. Data integrity indicates whether a dashboard could provide information on data sources, collection methods, and representativeness [68].

Furthermore, the “system capabilities” criterion, which involves dashboard features and capabilities, was regarded as a separate criterion for evaluating dashboards in the present study. To design a dashboard, functional and nonfunctional requirements should be taken into consideration. The functional requirements of dashboards denote the key functions of a system related to operations carried out or facilitated using that system. On the other hand, nonfunctional requirements are a set of specifications that are not directly related to users' tasks but could improve its functionality [9, 70].

Finally, it can be acknowledged that both quantitative and qualitative methods play a significant role in technology development and progress. While quantitative methods have some advantages, such as cost-effectiveness and higher suitability for studies with a large sample size, qualitative methods (e.g., think aloud) are beneficial for providing details about problems to which quantitative methods do not commonly apply [57]. Additionally, qualitative data analysis of user's behaviors and routines and a variety of other information are essential to deliver a product that actually fits into a user's needs or desires [71]. A combination of qualitative and quantitative approaches is suggested to appropriately measure the usability of technologies [57].

## 5. Strengths and Limitations

Since no study has yet designed a tool for evaluating usability of dashboards in healthcare, in this systematic review, a comprehensive analysis was carried out to remark usability evaluation criteria for dashboards. The usability evaluation criteria that could be used for dashboards were extracted by investigating 29 questionnaires used in previous available studies. However, there are limitations with the current study. First of all, although these studies provided a foundation for conducting our review and suggesting relevant criteria, further study is required to investigate the power of suggested criteria in practice. However, we have designed such a study to address the limitation noted. Second, this review only focused on quantitative studies and usability questionnaires, while qualitative approaches could help to provide a more robust construction for dashboard evaluation. However, we made an attempt to provide a basis for researchers who aim to measure different aspects of dashboards quantitatively, which is a well-used and common evaluation approach. In addition, we focused on English published literature, and we might have missed some relevant studies published in non-English languages.

## 6. Conclusion

Dashboards, as data management tools, play a crucial role in the decision-making and management of clinical and administrative data; therefore, they should be free of any usability-related problems. In this study, by reviewing the existing questionnaires used for the usability evaluation of dashboards, some criteria were suggested for evaluating dashboards, including usefulness, operability, learnability, ease of use, suitability for tasks, improvement of situational awareness, satisfaction, user interface, content, and system capabilities. When choosing criteria for the usability evaluation of dashboards, the study objectives, dashboard features and capabilities, and context of use should be taken into consideration.

## Figures and Tables

**Table 1:** The keywords used in the search strategy.

#1	Usability OR Testing OR evaluation OR Assessment OR satisfaction
#2	Dashboard OR “electronic whiteboard”
#3	Questionnaire OR Scale OR “Surveys and Questionnaires”
#1 AND #2 AND #3	

**Table 2:** Characteristics of the questionnaires.

Questionnaire name	Number of questions; scoring	Subscales	GR
TAM [25–29]	15; 5-point Likert scale (“strongly disagree” to “strongly agree”)	(i) Perceived ease of use (ii) Perceived usefulness	(i) 0.98 (usefulness) (ii) 0.94 for (ease of use)
UTAUT [26, 30]	21; 7-point Likert scale (“strongly disagree” to “strongly agree”)	(i) Mechanical ease of use (ii) Cognitive ease of use (iii) Emotional difficulty (iv) Decision-aiding effectiveness	0.91
SUS [31–40]	10; 5-point Likert scale (“strongly disagree” to “strongly agree”)	—	0.91
SART [34, 41, 42]	10; 7-point Likert scale (“strongly disagree” to “strongly agree”)	(i) Instability representation (ii) Complexity representation (iii) Variability representation (a) Arousal support (b) Concentration support (c) Spare mental capacity support (d) Division of attention (e) Information quantity (f) Information quality (g) Familiarity with dashboard	0.92
Health-ITUES [27, 34, 43]	20; 5-point Likert scale (“strongly agree” to “strongly disagree”) and N/A	(i) Quality of work life (ii) Perceived usefulness (iii) Perceived ease of use (iv) User control	0.81 to 0.95
PSSUQ [32]	19; 7-point Likert scale (“strongly agree” to “strongly disagree”) and N/A	(i) System usefulness (ii) Information quality (iii) Interface quality	0.96
QUIS [35, 44]	27; 10-point Likert scale (several adjectives positioned from negative to positive) and N/A	(i) Overall reaction to the software (ii) Screen (iii) Terminology and system information (iv) Learning (v) System capabilities	0.94
CSUQ [45]	19; 7-point Likert scale (“strongly agree” to “strongly disagree”) and N/A	(i) System usefulness (ii) Information quality (iii) Interface quality	0.95
EUCS [46]	12; 5-point Likert scale (“very strong” to “very dissatisfied”) and N/A	(i) Accuracy (ii) Content (iii) Ease of use (iv) Format (v) Timeliness	0.95
DATUS [47]	20; 7-point Likert scale (“strongly agree” to “strongly disagree”) and N/A	(i) Effectiveness (ii) Efficiency (iii) Satisfaction (iv) Learnability (v) Accessibility (vi) Appropriate recognizability (vii) User interface aesthetics (viii) Operability	NR
Batley et al. [48]	8; 5-point Likert scale (“strongly agree” to “strongly disagree”) and N/A	—	NR
Hertzum [49]	21; 7-point Likert scale (“strongly agree” to “strongly disagree”) and N/A	—	NR
Pickering et al. [50]	10; 5-point Likert scale (“strongly agree” to “strongly disagree”) and N/A	—	0.87 to 0.91
Tan et al. [51]	12; 5-point Likert scale (“strongly agree” to “strongly disagree”) and N/A	(i) Overall user satisfaction (ii) Usage frequency (iii) System quality (e.g., speed, ease of use, and stability) (iv) System information quality (e.g., accuracy and relevancy of data) (v) Impact on work efficiency (vi) Impact on care quality (e.g., effectiveness and safety)	NR
Lai et al. [52]	15; 5-point Likert scale (“strongly agree” to “strongly disagree”) and N/A	—	0.87 to 0.91

TAM: Technology Acceptance Model; UTAUT: Unified Theory of Acceptance and Use of Technology; SUS: System Usability Scale; SART: Situation Awareness Rating Technique; Health-ITUES: Health Information Technology Usability Evaluation Scale; PSSUQ: Post-Study System Usability Questionnaire; QUIS: Questionnaire for User Interaction Satisfaction; CSUQ: Computer System Usability Questionnaire; EUCS: End-User Computing Satisfaction Model; DATUS: Dashboard Assessment Usability Model; GR: Global Reliability; NR: not reported.

**Table 3:** Usability evaluation criteria for dashboards.

Criteria	Subcriteria		Questionnaire
Usefulness	(i) Perform tasks more effectively using dashboards (ii) The effectiveness of the information displayed by the dashboard in completing the tasks of users (iii) Better control of activities and improvement of job performance (iv) Perform tasks faster using the dashboard (v) Dashboard has been designed to maximize efficiency		Health-ITUES PSSUQ CSUQ TAM
Operability	(i) Displaying the level of details of data using a hierarchical structure (ii) Report formats should include relevant data dimensions. Easy to identify, select, and view data dimensions (iii) Data should be accessible at different levels of aggregation. Visibility and availability of filters applied to the data (iv) The speed of system recovery when the user makes a mistake (v) User control		DATUS
Learnability	(i) Easy to learn (ii) The speed of learning to use the dashboard (iii) Clarity of information (such as online help and on-screen messages) (iv) Comprehensibility of the information displayed by the dashboard		QUIS
Ease of use	(i) Easy to use dashboard to perform tasks (ii) Use the dashboard without needing help or guidance from others (iii) It is easy to find the required information in the dashboard		EUCS Health-ITUES TAM
Suitability for tasks	(i) Ability to support users' daily activities (ii) Fit and organize the information on the screen to the user's tasks (iii) Compatibility and organization of information on the screen with the user's tasks (iv) The possibility of setting the way of displaying software outputs (reports) with user tasks		DATUS
Improving situational awareness	(i) Instability representation (ii) Complexity representation (iii) Variability representation (iv) Arousal support (v) Concentration support (vi) Spare mental capacity support (vii) Division of attention		SART
Satisfaction	(i) Overall satisfaction in using the dashboard (ii) Feel comfortable using the dashboard (iii) Satisfaction with the dashboard user interface (iv) Satisfaction with dashboard features and capabilities		QUIS
User interface	(i) Visualization tools	(i) Color coding (ii) Data visualization: histogram plot, pie chart, bar graphs, gauges, data table, geographic maps	DATUS PSSUQ
	(ii) Interaction	(i) Overview, zoom, filter, details-on-demand, control level of details, redo/undo, navigation and querying, data set reduction, customizable, drill up, and drill down	DATUS PSSUQ CSUQ
Content	(i) Information quantity provided	(i) The quantity of information is appropriate for performing tasks	SART EUCS
	(ii) Information quality provided	(i) Quality of information: accuracy, up-to-date, comprehensive, relevant	SART PSSUQ CSUQ
System capabilities	(i) Having all the functions and features expected by users (ii) Having the right speed (iii) Integration of all expected functions in the system		QUIS

## Data Availability

All data generated or analyzed during this study are included in this published article. The data used to support the findings of this study are included within the supplementary information file(s).

## Supplementary Materials

Supplementary Materials Table A1: appraisal result of study quality for quasiexperimental studies using the JBI-M AStARI. Table A2: appraisal result of study quality for the RCT using the JBI-MAStARI. Table A3: examining dashboard evaluation criteria in included articles. Table A4: dimensions to measure usability discarded from the model.
