# Supplementary material for: Usability Evaluation of Dashboards: A Systematic Literature Review of Tools
Source: Biomed Res Int. 2023 Feb 22;2023:9990933. doi: 10.1155/2023/9990933 (PMC9977530; doi:10.1155/2023/9990933)
Supplement: Supplementary Materials — Table A1: appraisal result of study quality for quasiexperimental studies using the JBI-M AStARI. Table A2: appraisal result of study quality for the RCT using the JBI-MAStARI. Table A3: examining dashboard evaluation criteria in included articles. Table A4: dimensions to measure usability discarded from the model. [file 9990933.f1.zip › Appendix A (1).docx]

Appendix A
Table A1. Appraisal result of study quality for quasi-experimental studies using the JBI-MAStARI

| Citation | Q1 | Q2 | Q3 | Q4 | Q5 | Q6 | Q7 | Q8 | Q9 | Score |
| --- | --- | --- | --- | --- | --- | --- | --- | --- | --- | --- |
| Batley et al | Y | N | N | N | Y | Y | Y | Y | Y | 6 |
| Hertzum | Y | N | N | N | Y | Y | Y | Y | Y | 6 |
| Taneva et al | Y | N | N | N | Y | Y | Y | Y | Y | 6 |
| Dolan et al | Y | N | N | N | Y | Y | Y | Y | Y | 6 |
| Tan et al | Y | N | N | N | Y | Y | Y | Y | Y | 6 |
| De Croon et al | Y | N | N | N | Y | Y | Y | Y | Y | 6 |
| Franklin et al | N | Y | N | N | Y | Y | Y | Y | N | 5 |
| Lee et al | Y | N | N | N | Y | Y | Y | Y | Y | 6 |
| Mlaver et al | Y | Y | N | N | Y | Y | Y | Y | Y | 7 |
| Schall et al | Y | Y | N | N | Y | Y | Y | Y | Y | 7 |
| Dowding et al | Y | Y | N | N | Y | Y | Y | Y | Y | 7 |
| Martinez et al | Y | Y | N | N | Y | Y | Y | Y | Y | 7 |
| Rouhani et al | Y | Y | N | N | Y | Y | Y | Y | Y | 7 |
| Yoo et al | Y | Y | N | N | Y | Y | Y | Y | Y | 7 |
| Barbeito, and Segall | Y | Y | N | N | Y | Y | Y | Y | Y | 7 |
| Dowding et al | Y | Y | N | N | Y | Y | Y | Y | Y | 7 |
| Wu et al | Y | Y | N | N | Y | Y | Y | Y | Y | 7 |
| Antunes | Y | Y | N | N | Y | Y | Y | Y | Y | 7 |
| Bersani et al | Y | Y | N | N | Y | Y | Y | Y | Y | 7 |
| Fischer et al | Y | Y | N | N | Y | Y | Y | Y | Y | 7 |
| Lagha et al | Y | Y | N | N | Y | Y | Y | Y | Y | 7 |
| Alvarado et al | Y | Y | N | N | Y | Y | Y | Y | Y | 7 |
| Roa Romero et al | Y | Y | N | N | Y | Y | Y | Y | Y | 7 |
| Xiao et al | Y | Y | N | N | Y | Y | Y | Y | Y | 7 |
| Khanbhai et al | Y | Y | N | N | Y | Y | Y | Y | Y | 7 |
| Randell et al | Y | Y | N | N | Y | Y | Y | Y | Y | 7 |

(1) is it clear in the study what is the ‘cause’ and what is the ‘effect’ (for sample, there is no confusion about which variable comes first); (2) were the participants included in any comparisons similar; (3) were the participants included in any comparisons receiving similar treatment/care, other than the exposure or intervention of interest; (4) was there a control group; (5) were there multiple measurements of the outcome both pre and post the intervention/exposure; (6) was follow-up completed and if not, were differences between groups in terms of their follow-up adequately described and analyzed; (7) were the outcomes of participants included in any comparisons measured in the same way; (8) were outcomes measured in a reliable way; and (9) was appropriate statistical analysis used?

Table A2. Appraisal result of study quality for the RCT using the JBI-MAStARI

| Citation | Q1 | Q2 | Q3 | Q4 | Q5 | Q6 | Q7 | Q8 | Q9 | Q10 | Q11 | Q12 | Q13 | Score |
| --- | --- | --- | --- | --- | --- | --- | --- | --- | --- | --- | --- | --- | --- | --- |
| Koch et al | Y | Y | Y | Y | Y | Y | U | Y | Y | Y | Y | Y | Y | 12 |
| Pickering et al | U | Y | U | N | N | Y | Y | Y | Y | Y | Y | Y | Y | 9 |
| Lai et al | U | Y | Y | N | N | Y | Y | Y | Y | Y | Y | Y | Y | 10 |

(1) was true randomization used for assignment of participants to treatment groups; (2) was allocation to treatment groups concealed, treatment groups similar at the baseline; (3) were participants blind to treatment assignment, (4) were those delivering treatment blind to treatment assignment; (5) were outcomes assessors blind to treatment assignment; (6) were treatment groups treated identically other than the intervention of interest; (7) was follow-up completed and if not, were differences between groups in terms of their follow-up adequately described and analyzed; (8) were participants analyzed in the groups to which they were randomized; (9) were outcomes measured in the same way for treatment groups; (10) were outcomes measured in a reliable way; (11) was appropriate statistical analysis used; (12) was the trial design appropriate; and (13) were any deviations from the standard RCT design (individual randomization, parallel groups) accounted for in the conduct and analysis of the trial?
