# Supplementary material for: Usability Evaluation of Dashboards: A Systematic Literature Review of Tools
Source: Biomed Res Int. 2023 Feb 22;2023:9990933. doi: 10.1155/2023/9990933 (PMC9977530; doi:10.1155/2023/9990933)
Supplement: Supplementary Materials — Table A1: appraisal result of study quality for quasiexperimental studies using the JBI-M AStARI. Table A2: appraisal result of study quality for the RCT using the JBI-MAStARI. Table A3: examining dashboard evaluation criteria in included articles. Table A4: dimensions to measure usability discarded from the model. [file 9990933.f1.zip › Appendix B (1).docx]

**Appendix B**

**Table A3: Examining dashboard evaluation criteria in included articles**

| **Evaluation questionnaire** | **Evaluation outcome** | **Country** | **Reference(year)** |
| --- | --- | --- | --- |
| Self-made questionnaire | Feature and functionality | Lebanon | Batley, et al (2011) |
| Self-made questionnaire | Satisfaction | Denmark | Hertzum, (2011) |
| TAM | Usefulness, satisfaction | Switzerland | Taneva, et al (2011) |
| SART | Effectiveness | USA | Koch, et al (2013) |
| UTAUT | Ease of use, effectiveness | Germany | Dolan, et al (2013) |
| Self-made questionnaire | Satisfaction, efficiency, effectiveness | Singapore | Tan, et al (2013) |
| SUS | Satisfaction | Belgium | De Croon, et al (2015) |
| Self-made questionnaire | Effectiveness | USA | Pickering, et al (2015) |
| SART | Efficiency, effectiveness | USA | Franklin, et al (2017) |
| UTAUT  TAM | Usefulness, ease of use, effectiveness | South Korea | Lee, et al (2017) |
| Health-ITUES | Ease of use, usefulness | USA | Mlaver, et al (2017) |
| SUS, PSSUQ | Satisfaction, usefulness | USA | Schall, et al (2017) |
| QUIS, SUS | Satisfaction | United Kingdom | Dowding, et al(2018) |
| CSUQ | Ease of use, satisfaction | USA | Martinez, et al (2018) |
| EUCS | Satisfaction | Iran | Rouhani, et al (2018) |
| SUS. SART | Satisfaction, efficiency, effectiveness | South Korea | Yoo, et al (2018) |
| QUIS | Satisfaction | USA | Barbeito, et al (2019) |
| QUIS, SUS | Satisfaction | USA | Dowding, et al (2019) |
| SUS | Satisfaction | USA | Wu, et al (2019) |
| DATUS | Satisfaction, effectiveness, efficiency | Portugal | Antunes, (2020) |
| Health-ITUES | Ease of use, usefulness | USA | Bersani, et al (2020) |
| TAM | Ease of use, usefulness | USA | Fischer, et al (2020) |
| SUS | Satisfaction | USA | Richter Lagha, et al(2020) |
| TAM | Ease of use, usefulness | United Kingdom | Alvarado, et al (2021) |
| SUS | Satisfaction | Germany | Roa Romero, et al (2021) |
| SUS | Satisfaction | Canada | Xiao, et al (2021) |
| SUS | Satisfaction | United Kingdom | Khanbhai, et al (2022) |
| Self-made questionnaire | efficiency | Taiwan | Lai, et al (2022) |
| TAM | Usefulness, ease of use | United Kingdom | Randell, et al (2022) |
