# Supplementary material for: Usability Evaluation of Dashboards: A Systematic Literature Review of Tools
Source: Biomed Res Int. 2023 Feb 22;2023:9990933. doi: 10.1155/2023/9990933 (PMC9977530; doi:10.1155/2023/9990933)
Supplement: Supplementary Materials — Table A1: appraisal result of study quality for quasiexperimental studies using the JBI-M AStARI. Table A2: appraisal result of study quality for the RCT using the JBI-MAStARI. Table A3: examining dashboard evaluation criteria in included articles. Table A4: dimensions to measure usability discarded from the model. [file 9990933.f1.zip › Appendix C (1).docx]

**Appendix C**

**Table A4**: Dimensions to measure usability discarded from the model

| **Dimension** | **From questionnaire** | **Acceptance** | **Reason to discard** |
| --- | --- | --- | --- |
| Accessibility | DATUS | × | To be measured in ease of use |
| Effectiveness | DATUS | × | To be measured in Usefulness |
| Efficiency | DATUS | × | To be measured in Usefulness |
| Operability | DATUS | ✓ | - |
| User interface aesthetics | DATUS  PSSUQ | ✓ | - |
| Suitability for Tasks | DATUS | ✓ | - |
| Information quantity provided | SART | ✓ | - |
| Information quality provided | SART  PSSUQ | ✓ | - |
| Accuracy | EUCS | × | To be measured in content. |
| Content | EUCS | ✓ | - |
| Ease of use | EUCS  Health-ITUES  TAM | ✓ | - |
| Format | EUCS | × | To be measured in system capabilities |
| Timeliness | EUCS | × | To be measured in content |
| Usefulness | Health-ITUES  PSSUQ  CSUQ  TAM | ✓ | - |
| Information quality | CSUQ | ✓ | - |
| Interface Quality | CSUQ | ✓ | - |
| Screen | QUIS | × | To be measured in user interface |
| Learning | QUIS | ✓ | - |
| System Capabilities | QUIS | ✓ | - |
| Satisfaction | QUIS | ✓ | - |
| Improving situational awareness | SART | ✓ | - |
| User control | Health-ITUES | × | To be measured in operability |
